# Supplementary material for: Mechanisms governing the pioneering and redistribution capabilities of the non-classical pioneer PU.1
Source: Nat Commun. 2020 Jan 21;11:402. doi: 10.1038/s41467-019-13960-2 (PMC6972792; doi:10.1038/s41467-019-13960-2)
Supplement: Supplementary file 7 — Source data [file 41467_2019_13960_MOESM7_ESM.zip › Source_Data/FigureS3/FigureS3J_MotifScanOutput/homerResults/motif3.similar.html]

motif3

## Information for motif3

C
A
T
G
C
A
G
T
A
G
C
T
A
G
T
C
G
T
C
A
T
C
G
A
C
T
G
A
A
C
T
G
A
C
G
T
A
G
T
C
G
A
T
C
G
C
T
A
  
Reverse Opposite:  

C
G
A
T
C
T
A
G
T
C
A
G
T
G
C
A
T
G
A
C
G
A
C
T
A
G
C
T
C
A
G
T
T
C
A
G
T
C
G
A
G
T
C
A
G
T
A
C
  

|  |  |
| --- | --- |
| p-value: | 1e-426 |
| log p-value: | -9.816e+02 |
| Information Content per bp: | 1.575 |
| Number of Target Sequences with motif | 4372.0 |
| Percentage of Target Sequences with motif | 33.13% |
| Number of Background Sequences with motif | 5338.0 |
| Percentage of Background Sequences with motif | 14.62% |
| Average Position of motif in Targets | 391.2 +/- 353.2bp |
| Average Position of motif in Background | 318.3 +/- 202.0bp |
| Strand Bias (log2 ratio + to - strand density) | -0.0 |
| Multiplicity (# of sites on avg that occur together) | 1.23 |
| Motif File: | file (matrix) reverse opposite |

### Similar de novo motifs found

|  |  |  |  |  |  |  |  |
| --- | --- | --- | --- | --- | --- | --- | --- |
| Rank | Match Score | Redundant Motif | P-value | log P-value | % of Targets | % of Background | Motif file |
| 1 | 0.975 | C G A T C A T G T C A G T G C A T G A C G A C T A G C T C A G T C T A G T C G A G T C A G A T C G A C T | 1e-396 | -912.494298 | 41.29% | 21.61% | motif file (matrix) |
| 2 | 0.951 | C T A G C A G T A G C T A G T C G T C A T C G A C T G A C A T G C A T G A G T C G A T C | 1e-386 | -890.908120 | 38.83% | 19.81% | motif file (matrix) |
| 3 | 0.965 | C G A T C T A G C T A G T G C A A T G C A G C T A G C T A C G T C T A G C T A G G T C A G T A C G A C T A G T C | 1e-335 | -772.663314 | 43.59% | 24.99% | motif file (matrix) |
| 4 | 0.930 | G C A T A G T C T G C A C T G A T C G A C T A G A C T G G A T C G A T C G C T A | 1e-283 | -652.629245 | 47.90% | 30.24% | motif file (matrix) |
| 5 | 0.937 | G A C T C T A G C T A G G T C A T G A C A G C T A C G T C A G T C T A G | 1e-253 | -582.836568 | 49.70% | 32.83% | motif file (matrix) |
| 6 | 0.846 | A T G C A G C T A G C T A C G T C T A G G C T A G T C A A G T C | 1e-206 | -474.716194 | 56.61% | 41.08% | motif file (matrix) |
| 7 | 0.813 | A T G C A G C T A G C T A C G T T C A G C G T A G C T A T A G C G C A T | 1e-204 | -471.682595 | 46.90% | 31.85% | motif file (matrix) |
| 8 | 0.824 | T A G C A G C T A G C T A C G T T A C G C G T A G C T A T A G C G C T A T A C G | 1e-203 | -468.980093 | 57.56% | 42.11% | motif file (matrix) |
| 9 | 0.765 | C G A T C T A G C T G A T A G C A T G C A G C T G A C T A C G T | 1e-154 | -355.075167 | 42.51% | 29.69% | motif file (matrix) |
| 10 | 0.786 | A T G C A G C T A C G T A C G T A C T G C G T A G T A C | 1e-118 | -273.721473 | 57.15% | 45.37% | motif file (matrix) |
| 11 | 0.762 | A C T G A C T G G T C A G T A C A C G T A G C T A C G T | 1e-87 | -201.060107 | 40.38% | 30.78% | motif file (matrix) |
